# Behavioral and Personal Characteristics Associated With Risk of SARS-CoV-2 Infection in a Spanish University Cohort

**DOI:** 10.1093/aje/kwad086

**Published:** 2023-04-12

**Authors:** Fares Amer, Mario Gil-Conesa, Silvia Carlos, Arturo H Ariño, Francisco Carmona-Torre, Miguel A Martínez-González, Alejandro Fernandez-Montero

**Keywords:** behavior, COVID-19, education, university

## Abstract

The aim of this study was to analyze the life habits and personal factors associated with increased severe acute respiratory syndrome coronavirus 2 (SARS-CoV-2) risk in a university environment with in-person lectures during the coronavirus disease 2019 (COVID-19) pandemic. To our knowledge, there are no previous longitudinal studies that have analyzed associations of behavioral and personal factors with the risk of SARS-CoV-2 infection on an entire university population. A cohort study was conducted in the 3 campuses of the University of Navarra between August 24, 2020, and May 30, 2021, including 14,496 students and employees; the final sample included 10,959. Descriptive and multivariate-adjusted models were fitted using Cox regression. A total of 1,032 (9.4%) participants were diagnosed with COVID-19 (879 students and 153 employees), almost 50% living with their families. COVID-19 was associated with living in college or residence (hazard ratio (HR) = 1.96, 95% CI: 1.45, 2.64), motor transportation (HR = 1.35, 95% CI: 1.14, 1.61), South American origin (HR = 1.43, 95% CI: 1.20, 1.72), and belonging to Madrid’s campus (HR = 3.11, 95% CI: 2.47, 3.92). International students, especially from Latin America, mostly lived in university apartments or shared flats and cohabited with 4–11 people. Living in a big city (Madrid), was a significant risk factor.

## Abbreviations

CI: confidence interval
COVID-19: coronavirus disease 2019
FFP: filtering face piece
HR: hazard ratio
PCR: polymerase chain reaction
SARS-CoV-2: severe acute respiratory syndrome coronavirus 2

The global fight against coronavirus disease 2019 (COVID-19) since March 2020 has led populations to adapt and develop new lifestyle habits to prevent infection, such as social distancing and use of masks and hand sanitizers. Every business or institution has implemented new rules and physically adapted their premises to promote a safe return to normality and the improvement of the economic situation.

Education is one of the most important pillars of any society, and not even a pandemic should stop students from adequate development. The COVID-19 pandemic led educational institutions to immediately develop ways to get teachers and students in touch to keep learning, while health authorities were trying to develop and adapt more accurate and less restrictive measures. In Spain, the lockdown was imposed on March 14, 2020, and from that point, the aim of educational institutions was to go back to normal operation in the next academic year, starting in September 2020. While most educational institutions remained closed and set up online learning methods, a few bet on implementing extraordinary measures to remain open and had a solid plan to prevent the increase of COVID-19 cases, which have been demonstrated to be effective (1).

Since there were no guides about how to proceed in such a situation, educational institutions around the world focused on developing adapted measures, in accordance with health authorities’ indications, with the aim of getting students back to in-person lectures. Regarding universities, some initiatives were successfully developed to achieve this objective, some were based on behavioral guidelines (Prepara2, Back2BU) (2), and others even included binding contracts with corrective measures for students (e.g., “Best for all” agreement (3)). The monitoring in each program included, along with other risk factors, polymerase chain reaction (PCR) testing, close contact tracing, warnings, robust quarantine measures, social distancing, and use of protective equipment adherence (2).

With all of the preventive measures implemented at the beginning of the academic year of 2020–2021 in September 2020, at the University of Navarra (1) we aimed to study life habits and personal factors associated with an increased severe acute respiratory syndrome coronavirus 2 (SARS-CoV-2) risk among students and employees, 2 populations with highly differentiated characteristics and lifestyles, in a university environment with in-person lectures during the COVID-19 pandemic.

## METHODS

### Study design

This is a cohort study, conducted at the 3 campuses of the University of Navarra in Spain (Pamplona, Gipuzkoa, and Madrid), between August 2020 and May 2021. The entire university population was invited to participate by completing a baseline questionnaire at the beginning of the academic year, and the incidence of SARS-CoV-2 infection was analyzed by mass PCR testing.

### Participants

**Figure 1:**
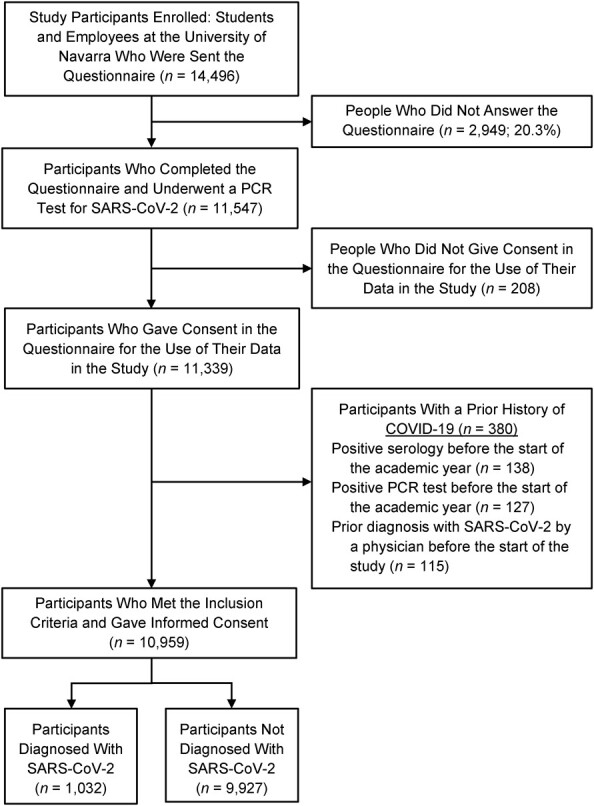
Flowchart of the sample studied during the coronavirus disease 2019 (COVID-19) pandemic, University of Navarra, Spain, during the academic year 2020–2021. PCR, polymerase chain reaction; SARS-CoV-2, severe acute respiratory syndrome coronavirus 2.

The entire population of 14,496 participants (students and employees) from the 3 campuses of the University of Navarra (Pamplona, Gipuzkoa, and Madrid) were eligible and were sent the study questionnaire. Participants were either students or employees, and they indicated their primary status in the questionnaire, although there might be employees taking courses at the university. Those who completed the questionnaire and gave their consent in the first question were included in the study (11,547; response rate: 79.7%). Participants who reported a SARS-CoV-2 infection with a positive PCR test, presented a positive serology, or were diagnosed with COVID-19 by a physician prior to the start of the academic year were excluded. Ultimately, data from 10,959 participants were analyzed (Figure 1).

### Baseline questionnaire

The baseline questionnaire included questions on sociodemographic characteristics, type of residence during the academic year (university apartment, including participants who shared a flat or who lived alone and who did not live in their family home; college or residence; family home), number of people with whom they lived, living with children at home, origin country, hours of daily socializing, living with pets, and transportation (motor transportation included private motorized transport, car or motorbike, shared or not; public transportation included bus, metro, train or taxi, walking, cycling, or electric scooter). Questions on preventive measures were included: handwashing with soap, use of alcohol-based solutions, social distancing, gloves, and use of masks (filtering face piece (FFP; grades 2 or 3), surgical mask, or cloth mask). Data were collected on weight and height, as well as smoking status. Finally, participants were asked about comorbidities (diabetes, cancer, cardiovascular disease, chronic lung disease, impaired coagulation, hypertension, immunosuppression, chronic kidney disease, and chronic liver disease) and treatment.

### PCR testing

Random PCR testing was done using the following strategy: 1) mandatory PCR testing for all students and employees at the beginning of the academic year in August 2020 and after Christmas 2020; 2) PCR testing for symptomatic individuals and close contacts; 3) weekly random sampling during 8 weeks in the months of October to December, excluding holidays, and stratified by the 3 academic centers, based on the weekly incidence of SARS-CoV-2 infection in August 2020 (total population size was 14,496, a 95% confidence level and a margin of error of 0.6 generated a required sample size of 262 individuals, and a total of 1,706 PCR tests were performed (mean, 226 (standard deviation, 55.4) per week)); and 4) extra, random PCR testing after the Easter holiday that included 2,570 participants. The back-to-in-person plan is explained in detail elsewhere (1). A total of 34,000 PCR tests were performed and analyzed in 2 different laboratories: 1) At the Centro de Investigación Médica Aplicada (CIMA), mass sampling for the entire university population was performed at the beginning of the academic year and after vacation periods. In addition, CIMA analyzed all samples from university-bound close contacts, random subjects on a weekly schedule, and population with mild symptoms. 2) At the Clínica Universidad de Navarra (CUN), SARS-CoV-2 samples from the population showing symptoms compatible with COVID-19 were analyzed.

Those individuals who, during the period of the study, submitted external medical reports of positive SARS-CoV-2 PCR or antigen tests and their corresponding PCR or antigen test made in a health-care center to the COVID Area were also considered positive cases. In any case, a PCR test was always made to follow up on the infection.

### Statistical analysis

An independent samples *t* test was used to compare continuous variables such as age or body mass index, while a χ^2^ test was used for categorical variables.

Hazard ratios (HRs) and their 95% confidence intervals (CIs) were estimated using Cox regression models. For each participant, follow-up was from the beginning of the academic year to the end of the academic year or the first positive SARS-CoV-2 PCR test. A positive PCR test was considered as the outcome variable and being a student or employee as the main independent variable. The variables related to the situation and habits of the members of the university were considered as independent variables. Analyses of a subset of covariates related to the situation and habits of the university members (type of housing, transport, origin, campus, cohabitants) were also considered as independent variables.

Multivariate-adjusted models adjusted for sex, age, type of housing, employee or student, body mass index, smoking status, hours of socializing, comorbidities, cohabitant adults, cohabitant children, university campus, most commonly used form of transport, frequency of handwashing, hand sanitizer use, and mask use (cloth, surgical, or FFP (grades 2 or 3) masks), pets during the academic year, and region of origin. Although there were few missing values, in the multivariate models, multiple imputations were made for the variables body mass index, hours of socializing, comorbidities, type of housing, and the number of cohabitants, taking into account all other variables. To perform multiple imputation, we used the *mi impute* regress command in Stata (StataCorp LLC, College Station, Texas), using the Gaussian normal regression imputation method, with 20 repeated imputations). In the imputation method we used the same covariates used in the adjusted Cox regression along with the main independent variable itself and the outcome variable. The variables with the most missingness were body mass index (*n* = 1,092; 10%) and daily hours of socializing (*n* = 1,938; 17.7%).

Nelson-Aalen curves were used to describe the incidence of SARS-CoV-2 infection as a function of whether members were students or employees. Additionally, inverse probability weighting methods were applied to these curves to create adjusted curves. The same covariates used in the multivariate-adjusted Cox regression were used. After the weights were stabilized, the mean of the stabilized weights was equal to 1 and the standard deviation was 2.26.

All *P* values were 2-tailed; *P* < 0.05 was considered statistically significant. Analyses were performed using STATA, version 15.0 (StataCorp LLC).

This study followed the Strengthening of the Reporting of Observational Studies in Epidemiology (STROBE) reporting guidelines for cohort studies.

The study was conducted in compliance with the study protocol, the current version of the Declaration of Helsinki, and the local legal and regulatory requirements (approved by the University of Navarra Ethics committee: 2020.190 on October 30, 2020).

### Regions

In the regions variable, a synthesis was made of the countries of origin existing in the university, the regions were divided into the following categories: Spain; other European countries (Germany, Andorra, Austria, Belgium, Switzerland, Sweden, Russia, Romania, Czech Republic, United Kingdom, Portugal, Poland, Slovakia, France, Greece, the Netherlands, Hungary, Italy, Latvia, Liechtenstein, Luxembourg, Moldova, Norway, Ukraine, Serbia, Croatia, Bulgaria, Ireland, Denmark, Lithuania, Bosnia, and Herzegovina); and Latin America (Venezuela, Uruguay, Dominican Republic, Puerto Rico, Argentina, Bolivia, Brazil, Chile, Colombia, Costa Rica, Cuba, Ecuador, El Salvador, Perú, Guatemala, Haití, Honduras, Mexico, Nicaragua, Panama, and Paraguay). Other regions included Asia, Africa, North America, and Oceania. Asian countries: Vietnam, Taiwan, Singapore, Qatar, China, Saudi Arabia, United Arab Emirates, Philippines, Hong Kong, India, Indonesia, Iran, Lebanon, Sri Lanka, Kazakhstan, Republic of Korea, Syria, Turkey, and Japan; African countries: Tanzania, Nigeria, Uganda, Cape Verde, Cameroon, Democratic Republic of Congo, Kenya, Angola, Western Sahara, Algeria, Guinea, and Mauritania; North America: Canada and the United States; and Oceania: Australia.

### Dates of interest

- The questionnaire, along with the consent form, was sent prior to the PCR test at the beginning of the academic year: August 17, 2020.
- Testing began August 24, 2020.
- End of the mandatory testing fell at the beginning of the year: September 10, 2020.
- End of follow-up was May 30, 2021.

### Evidence before this study

We conducted a search of relevant literature in PubMed (National Center for Biotechnology Information, Bethesda, Maryland) through November 4, 2021, for studies carried out in universities, using the terms “(SARS-CoV-2 or COVID-19) AND (Universit^*^ or College or higher education)” without any language or date restrictions. We found few empirical studies showing objective diagnostic tests carried out in universities, most notably those carried out at the University of Wisconsin–Madison, at Boston University, and at Duke University.

## RESULTS

Out of 10,959 participants included in our study (77.7% students and 22.3% employees), 58% were female, and the mean age was 25.9 (95% CI: 25.7, 26.1) years. A total of 1,032 (9.4%) participants tested positive during the study. Students received 84.6% of the PCR tests, which means an average of 3.37 per student; 15.4% of PCR tests were of employees, with an average of 2.14 tests per employee. Almost half of the entire sample lived at home during the academic year (49.2%), compared with 32.4% who lived in a university flat, and 18.4% who lived in a college or residence. Most of the sample was from the Pamplona campus (85.5%), followed by Gipuzkoa (11%) and Madrid (3.5%). Regarding the country of origin, 83.8% were Spanish, and 11.4% Latin American, 2.5% from other European countries, and 2.4% from other regions. Most participants had 1–3 cohabitants (68.8%), with 15.1% having 11 or more cohabitants, 10.7% having 4–10 cohabitants, and 5.5% living alone; 77.3% reported not living with children during the academic year. The most frequent transportation was motor transport (46%), followed by public transport (30.7%) and walking (23.3%). Regarding daily hours of socializing, this was categorized into 5 groups and showed significant differences between students and employees; 78.7% of employees declared from 0–1 hours and 21.7% of students declared more than 3 hours. Finally, 94.4% of participants said that they did not have any comorbidities.

Although many characteristics showed significant differences between students and employees, the greatest differences were observed in the type of housing, region of origin, number of cohabitants, daily hours of socializing, type of transport most frequently used, and previous comorbidities. The baseline characteristics of the study participants according to the employee or student status can be seen in Table 1, and the same characteristics according to infection with SARS-CoV-2 can be seen in Table 2.

**Table 1:** Baseline Characteristics of University of Navarra Study Participants According to Student or Employee Status During the Academic Year, Campuses of Pamplona, Gipuzkoa, and Madrid, Spain, 2020–2021

**Variable**	**Students (*n* = 8,516)**		**Employees (*n* = 2,443)**		***P* Value**
	**No.**	**%**	**No.**	**%**	
Female sex	4,997	58.7	1,361	55.7	0.009
Age, years^a^	21.0 (3.2)		42.9 (11.6)		<0.001
BMI^a^^,^^b^	22.0 (3.3)		24.0 (3.8)		<0.001
Type of housing					<0.001
Family home	3,296	38.7	2,090	85.6	
University apartment	3,327	39.1	227	9.3	
University residence or hall	1,893	22.2	124	5.1	
University campus					<0.001
Pamplona	7,380	86.7	1,988	81.4	
Gipuzkoa	859	10.1	350	14.3	
Madrid	277	3.3	105	4.3	
Region of origin					<0.001
Spain	6,857	80.8	2,289	94.1	
Other European countries	223	2.6	48	2.0	
Latin America	1,175	13.8	71	2.9	
Other continents	233	2.7	24	1.0	
Cohabiting adults					<0.001
0	366	4.3	233	9.5	
1–3	5,745	67.5	1,785	73.1	
4–10	954	11.2	220	9.0	
≥11	1,444	17.0	205	8.4	
Cohabiting with children	1,500	17.6	989	40.5	<0.001
Daily hours of socializing					<0.001
0	899	12.8	917	46.1	
0.1–0.9	1,459	20.7	649	32.6	
1.0–1.9	1,905	27.1	261	13.1	
2.0–2.9	1,242	17.7	64	3.2	
≥3.0	1,527	21.7	98	4.9	
Most common transportation					<0.001
Walking and cycling	2,082	26.0	329	14.2	
Motor transport	3,223	40.2	1,533	66.0	
Public transportation	2,717	33.9	461	19.8	
Pets during the academic year					<0.001
None	7,396	86.8	2,043	83.6	
Dog	789	9.3	243	9.9	
Cat	198	2.3	90	3.7	
Other pet	133	1.6	67	2.7	
Prior diagnosis of comorbidities	293	3.4	325	13.3	<0.001
Smoker	867	10.2	201	8.2	0.004
Frequency of handwashing					<0.001
Never or sometimes	289	3.4	97	4.0	
Frequently	2,459	28.9	840	34.4	
Very frequently	5,768	67.7	1,506	61.6	
Use of the hand sanitizer					<0.001
Never or sometimes	616	7.2	227	9.3	
Frequently	2,500	29.4	937	38.4	
Very frequently	5,400	63.4	1,279	52.4	
Social distancing					<0.001
Never or sometimes	1,253	14.7	127	5.2	
Frequently	4,209	49.4	1,107	45.3	
Very frequently	3,054	35.9	1,209	49.5	
Use of gloves					<0.001
Never or sometimes	7,845	92.1	2,117	86.7	
Frequently	446	5.2	212	8.7	
Very frequently	225	2.6	114	4.7	
Use of cloth masks					<0.001
Never or sometimes	4,185	49.1	1,342	54.9	
Frequently	1,220	14.3	409	16.7	
Very frequently	3,111	36.5	692	28.3	
Use of surgical masks					0.002
Never or sometimes	1,739	20.4	536	21.9	
Frequently	1,784	20.9	570	23.3	
Very frequently	4,993	58.6	1,337	54.7	
Use of FFP2 or FFP3 masks					<0.001
Never or sometimes	5,382	63.2	1,761	72.1	
Frequently	1,482	17.4	328	13.4	
Very frequently	1,652	19.4	354	14.5	
Perception of correct implementation of preventive measures (scale: 1–10)^a^	8.2 (1.1)		8.5 (0.9)		<0.001
SARS-CoV-2 infection	879	10.3	153	6.3	<0.001

Abbreviations: BMI, body mass index; FFP2, filtering face piece, grade 2; FFP3, filtering face piece, grade 3; SARS-CoV-2, severe acute respiratory syndrome coronavirus 2.

^a^ Values are expressed as mean (standard deviation).

^b^ Weight (kg)/height (m)^2^.

**Table 2:** Baseline Characteristics of University of Navarra Study Participants According to SARS-CoV-2 Infection Status During the Academic Year, Campuses of Pamplona, Gipuzkoa, and Madrid, Spain, 2020–2021

**Variable**	**Negative (*n* = 9,927)**		**Positive (*n* = 1,032)**		***P* Value**
	**No.**	**%**	**No.**	**%**	
Female sex	5,775	76.9	583	56.5	0.30
Student status	7,637	76.9	879	85.2	<0.001
Age, years^a^	26.0 (11.1)		24.0 (9.3)		<0.001
BMI^a^^,^^b^	22.5 (3.5)		22.4 (3.4)		0.48
Type of housing					<0.001
Family home	5,051	50.9	335	32.5	
University apartment	3,118	31.4	436	42.3	
University residence or Hall	1,758	17.7	259	25.1	
University campus					<0.001
Pamplona	8,516	85.8	852	82.6	
Gipuzkoa	1,118	11.3	91	8.8	
Madrid	293	3.0	89	8.6	
Region of origin					<0.001
Spain	8,345	84.4	801	77.6	
Other European countries	251	2.5	20	1.9	
Latin America	1,052	10.6	194	18.8	
Other continents	240	2.4	17	1.6	
Cohabiting adults					<0.001
0	552	5.6	47	4.6	
1–3	6,885	69.4	645	62.5	
4–10	1,040	10.5	134	13.0	
≥11	1,443	14.5	205	20.0	
Cohabiting with children	0.70	6.6	0.45	2.2	0.24
Daily hours of socializing					0.012
0	1,670	20.4	146	17.6	
0.1–0.9	1,935	23.6	173	20.9	
1.0–1.9	1,945	23.7	221	26.7	
2.0–2.9	1,192	14.5	114	13.8	
≥3.0	1,451	17.7	174	21.0	
Most common transportation					0.005
Walking and cycling	2,219	23.7	192	19.8	
Motor transport	4,311	46.0	445	45.8	
Public Transportation	2,843	30.3	335	34.5	
Pets during the academic year					<0.001
None	8,504	85.7	935	90.6	
Dog	963	9.7	69	6.7	
Cat	270	2.7	18	1.7	
Other pet	190	1.9	10	1.0	
Prior diagnosis of comorbidities	9,358	94.3	971	94.1	0.23
Smoker	962	9.7	106	10.3	0.55
Frequency of handwashing					0.75
Never or sometimes	249	3.5	37	3.6	
Frequently	2,978	30.0	321	31.1	
Very frequently	6,600	66.5	674	65.3	
Use of the hand sanitizer					0.17
Never or sometimes	778	7.8	65	6.3	
Frequently	3,118	31.4	319	30.9	
Very frequently	6,031	60.8	648	62.8	
Social distancing					0.092
Never or sometimes	1,238	12.5	142	13.8	
Frequently	4,796	48.3	520	50.4	
Very frequently	3,893	39.2	370	35.9	
Use of gloves					0.19
Never or sometimes	9,009	90.8	953	92.3	
Frequently	603	6.1	55	5.3	
Very frequently	315	3.2	24	2.3	
Use of cloth mask					0.51
Never or sometimes	5,020	50.6	507	49.1	
Frequently	1,479	14.9	150	14.5	
Very frequently	3,428	34.5	375	36.3	
Use of surgical masks					0.81
Never or sometimes	2,054	20.7	221	21.4	
Frequently	2,130	21.5	224	21.7	
Very frequently	5,743	57.9	587	56.9	
Use of FFP2 or FFP3 mask					0.042
Never or sometimes	6,492	65.4	651	63.1	
Frequently	1,611	16.2	199	19.3	
Very frequently	1,824	18.4	182	17.6	
Perception of correct implementation of preventive measures (scale: 1–10)^a^	8.27 (1.0)		8.23 (1.1)		0.21

Abbreviations: BMI, body mass index; FFP2, filtering face piece, grade 2; FFP3, filtering face piece, grade 3; SARS-CoV-2, severe acute respiratory syndrome coronavirus 2.

^a^ Values are expressed as mean (standard deviation).

^b^ Weight (kg)/height (m)^2^.

**Figure 2:**
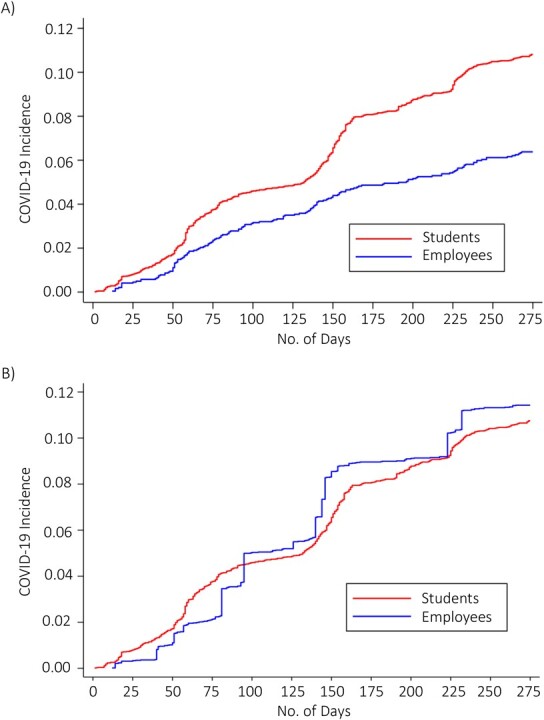
Nelson-Aalen plot of incidence of severe acute respiratory syndrome coronavirus 2 (SARS-CoV-2) infection in students and employees of the University of Navarra, Spain, in the academic year 2020–2021. Crude (A) and adjusted by inverse-probability weighting (B). COVID-19, coronavirus disease 2019.

Among the 1,032 infected participants, the mean age was 24 years, and 56% were women. There was a total of 879 positives among students, with an accumulative incidence at the end of the study period among students of 10.3%. Among employees, there were a total of 153 SARS-CoV-2 positive cases, with an accumulative incidence of 6.3% (Figure 2A). Applying inverse-probability weighting methods to the crude curve of Figure 2A revealed that the risk of SARS-CoV-2 infection was not related to the participants’ status (students or employees) (Figure 2B).

The observed crude HR of employees compared with students was 0.6 (95% CI: 0.50, 0.71); when adjusted for several factors, statistical significance was lost, showing this condition was not the cause of the higher cumulative incidence among students (Table 3). Although the cumulative incidence of SARS-CoV-2 for the staff group was 6.3%, when stratifying the staff groups into teachers and other staff, the rate was 4.6% for teachers and 7% for other university staff, although the differences were not significant due to the smaller sample size in each group (data not shown).

**Table 3:** Association Between Being a Student vs. Employee and SARS-CoV-2 Infection, Campuses of Pamplona, Gipuzkoa, and Madrid, Spain, Academic Year of 2021–2022

**Analysis**	**HR**	**95% CI**	***P* Value**
Crude model	0.60	0.50, 0.71	0.0001
Age- and sex-adjusted^a^	0.71	0.52, 0.95	0.021
Multivariate model^b^	1.07	0.79, 1.43	0.694

Abbreviations: CI, confidence interval; FFP2, filtering face piece, grade 2; FFP3, filtering face piece, grade 3; HR, hazard ratio; SARS-CoV-2, severe acute respiratory syndrome coronavirus 2.

^a^ Adjusted for sex and age.

^b^ Adjusted for sex, age, type of housing, body mass index, smoker, hours of socializing, comorbidities, cohabitant adults, cohabitant children, university campus, most commonly used form of transport, frequency of handwashing, frequency of hand sanitizer use, frequency of cloth mask use, frequency of surgical mask use, frequency of FFP2 or FFP3 mask use, pets during the academic year, and region of origin.

**Figure 3:**
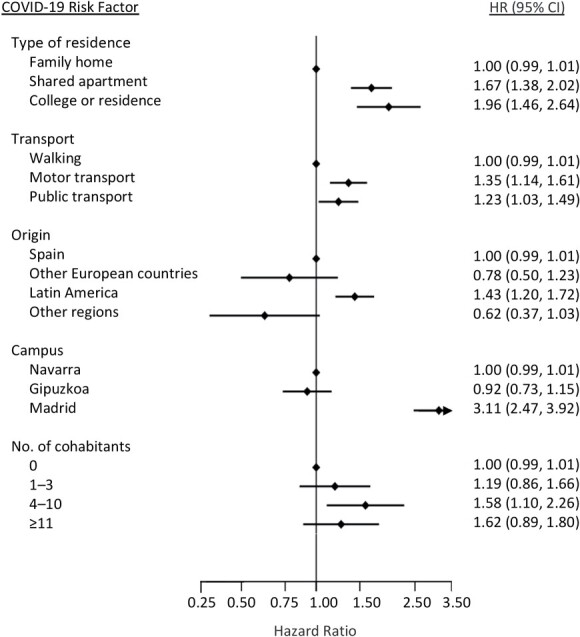
Hazard ratios (HRs) for social and behavioral factors shown to increase the risk of severe acute respiratory syndrome coronavirus 2 (SARS-CoV-2) infection at the University of Navarra, Spain, 2020–2021. Models adjusted for type of housing, employee vs. student status, age, sex, body mass index, smoking status, hours of socializing, comorbidities, cohabitant adults, cohabitant children, university campus, most commonly used form of transport, frequency of handwashing, frequency of hand sanitizer use, frequency of cloth mask use, frequency of surgical mask use, frequency of filtering facial piece (grades 2 or 3) mask use, pets during the academic year, and region of origin. CI, confidence interval; COVID-19, coronavirus disease 2019.

The independent factors that were shown to be relevant to the risk of SARS-CoV-2 infection were living in college or residence compared with living at home (HR = 1.96, 95% CI: 1.46, 2.64) and participants commuting around by motorized transport compared with walking/cycling (HR = 1.35, 95% CI: 1.14, 1.61). People from Latin America had an HR of 1.43 (95% CI: 1.20, 1.72); participants belonging to the Madrid campus had an HR of 3.11 (95% CI: 2.47, 3.92); and those living with 4–10 people had an HR of infection of 1.58 (95% CI: 1.10, 2.26) as compared with those who lived alone (Figure 3).

The preventive measures had generally very high reported adherence in all groups, so no differences were found in the multivariate models.

## DISCUSSION

This follow-up study analyzed the risk and lifestyle factors associated with being SARS-CoV-2–positive in a university population with 100% in-person classes and with pandemic preventive measures similar to those of other universities (2, 4–6).

Our study showed that when adjusting for multiple confounders and applying inverse-probability weighting methods, differences in the risk of SARS-CoV-2 infection between students and employees disappeared (Figure 2B). These findings put the focus of study on lifestyle and other sociodemographic risk factors, which although they are prevalent in students and make their risk of infection higher are not unique to students. A higher cumulative incidence of SARS-CoV-2 infection in students was observed overall, although it was not associated with older age, as it has been in other studies (7). Thus, it seems that the real risk factors are different habits—characteristics such as how individuals move, how they socialize, or where they live. Acting on these real risk factors is the key to preventing infection. In the present study, we carried out universal testing in both the groups, regardless of whether they were symptomatic or not.

Regarding the number of cohabitants, the results in the present study show that although only living with 4–10 cohabitants was shown to significantly increase the risk, the trend for all groups was for increased infection risk compared with living at the family home. Evidence shows that one of the most important determinants is cohabitant space; there may be a more than a 2-fold increased risk of infection if students live with a roommate or live at university residences (2). These data are consistent with the finding of an increased risk among those living in a flat or in a university residence. These results could be due to the fact that this type of cohabitation unit (4–10 cohabitants) is made up of student flats or small residences where the interaction is very close, as the number of inhabitants per square meter is higher than in flats with fewer inhabitants or in large university residences where each resident has his or her own individual room. A higher number of cohabitants in a confined space seems to be related to an increased infection risk. Additionally, students living in a shared flat or university residence have a less rule-bound lifestyle than in the family home and have more socializing hours. SARS-CoV-2 transmission is higher among household contacts than among nonhousehold contacts. This may be due to the closer and more prolonged interaction between these contacts than with social or work contacts (8), so in order to safely reopen a campus, the density of on-campus housing must be reduced (9).

The use of private motorized transportation and public transportation as a regular mode of commute showed a significantly increased risk of infection compared with walking, cycling, or using scooters. Sharing transport with a positive case has been shown to increase infection risk in other studies (8). It has also been shown that infection risk is considerably lower outdoors than indoors (10). Regarding commuting habits, in the study population, students reported more frequently walking and using public transport than did employees. This makes sense, in that despite using private transport less, students share this type of transport more frequently than do employees, generating a greater contagion risk in the use of public transport. When comparing the risk of contagion in employees and students who used motorized transport, students showed an HR of 1.35 (95% CI: 1.14, 1.61).

Among countries of origin, those from Latin America had an HR of 1.43 for infection at university after adjusting for multiple and lifestyle and other factors; this population has been shown to have a higher risk of SARS-CoV-2 in other studies (11, 12). This may be related to the virtual absence of Latin Americans in the employed population, as well as the absence of people from this group living with their relatives and in the family home.

Among university campuses, the risk of infection was 3 times higher in Madrid than in Pamplona. One of the factors that has been shown to contribute most to explaining the incidence at a university institution is the COVID-19 prevalence of the region where it is located, especially if it is an urban campus (2, 7, 13); this relationship is observed in all types of educational institutions (14), in both employees and students (15). During the study period, the reported cumulative incidence of infection over 14 days per 100,000 inhabitants was lower in Pamplona than in Madrid (1). Looking at the specific cases, a large proportion of the cases reported on the Madrid campus arose in 2 party venues in Madrid, a city and region whose policies concerning confinement and outdoor activities during the pandemic were notoriously less restrictive than in other parts of Spain (16). Nightlife is a risk activity for SARS-CoV-2 infection, and in large cities, access to mass gatherings in nightlife venues is greater. At the University of Navarra, the percentage of positive cases that was attributed to leisure activities in Navarra was 22% vs. 31% in Madrid (1)*.*

Other studies in university populations have shown very high rates of reported adherence to preventive measures, such as handwashing, wearing masks, or keeping social distance (13). Although these measures have not been shown to be a significant factor when adjusting for other variables, there is evidence for the effectiveness of preventive measures, including social distancing and wearing masks at social events, decreasing the risk in a university setting by 40% (4, 17). Cotton, surgical, and N95 respirator masks have been shown to be protective for SARS-CoV-2, although they have not proven to completely block its transmission (18). Different types were asked about in the questionnaire, as N95 masks have been shown to have the highest preventive efficacy, with an 80%–90% reduction in transmission, while cotton and surgical masks have been shown to block just over 50% of virus transmission. Handwashing and social distancing were also not significant factors in our study, although transmission is inversely related to the distance between subjects (18).

As was the case at the University of Navarra, in a review of the literature in university settings, infections did not usually occur on campus, and classmates or professors were not usually identified as the source (1, 2), nor has school or class size been associated with the number of SARS-CoV-2 cases (15), so the incidence of infection associated with educational settings is usually very low (1, 14, 19). Also no association has been found between SARS-CoV-2 transmission and in-person classes (4), despite the increase observed at the start of face-to-face classes in some areas of the United States (9). It should be noted that these increases are often observed after holiday periods and reopening of educational institutions (3, 5), so a strict policy of testing prior to arrival on campus is necessary.

The present study has some limitations. First, this study was based on self-reported data, including the lack of directly observed data on adherence to preventive measures and other baseline variables. However, participants belong to a university population with a high level of education that gives them adequate comprehension skills, and many of the study variables are sociodemographic. Baseline data were reported in the questionnaire while incidence data were collected from the University database in the COVID Area, where only positive diagnostic test results are collected. The analyses were carried out in a university population, and the results may not be generalizable to other types of populations. Also, retention of students in follow-up was over 99%, but we could not access losses to follow-up in the employee group.

Strengths of the present study include the high participation rate (79.7%) and the study design, which is a prospective cohort study, where participants who reported previous COVID-19 infection during an entire follow-up academic year were excluded. The diagnosis of SARS-CoV-2 infection was made by medical professionals through PCR testing, which has been shown to have a higher sensitivity than antigen testing (20) and ensures that data related to diagnosis and isolation are objective and not self-reported.

A university with exceptional measures to prevent SARS-CoV-2 infection can maintain 100% in-person classes and not generate incidence levels higher than those of the populations to which they belong. However, there are lifestyle and other factors specific to university communities that should be taken into account to try to reduce SARS-CoV-2 infections in university populations. University students are at higher risk mainly due to their circumstances in terms of housing, transport, or number of cohabitants. Adjusting for these factors, students were not shown to be a higher-risk population than employees.

### Added value of this study

We conducted a cohort study with questionnaires and PCR testing in a young university population, with follow-up from August 24, 2020, to May 30, 2021. During this period we observed 1,032 cases of SARS-CoV-2 among students and university employees. We were able to study risk factors for testing positive during the academic year, after adjusting for potential confounding variables. The variables that were associated with testing positive for SARS-CoV-2 were: the type of commuting, the campus of residence, number of people living together during the academic year, country of origin, and type of housing during the academic year. This is, to our knowledge, the first study of risk factors in a large university population with follow-up of an entire academic year.

### Implications of all the available evidence

Our findings show some risk factors for testing positive in a university setting, after adjusting for possible confounding variables. We did not find that student vs. employee status was an important determinant of risk for testing positive.
